# Prevalence and significance of indeterminate calcitonin values in patients with thyroid nodules: A systematic review and meta-analysis

**DOI:** 10.1007/s11154-023-09811-7

**Published:** 2023-05-31

**Authors:** Tommaso Piticchio, Francesco Frasca, Pierpaolo Trimboli

**Affiliations:** 1 Endocrinology Section, Department of Clinical and Experimental Medicine, Garibaldi Nesima Hospital, University of Catania, Catania, Italy; 2 Servizio di Endocrinologia e Diabetologia, Ente Ospedaliero Cantonale (EOC), Bellinzona, Switzerland; 3 Facoltà di Scienze Biomediche, Università della Svizzera Italiana (USI), Lugano, Switzerland

**Keywords:** Thyroid, Calcitonin, Medullary thyroid carcinoma, Systematic review, Meta-analysis

## Abstract

**Background:**

Although calcitonin (Ctn) measurement is recognized as the most accurate diagnostic test for medullary thyroid carcinoma (MTC), its routine execution is not universally accepted for several reasons, including the lack of recommendations for managing indeterminate Ctn values (ICV); such as 10-to-100 pg/mL. This study aimed to gather data on 1) the frequency of ICV among patients undergoing Ctn test and 2) the MTC rate among patients with ICV.

**Methods:**

This review was conducted according to the Meta-analyses Of Observational Studies in Epidemiology guidelines. PubMed and Cochrane databases were searched, with no language restrictions. The final search was completed on January 2023. Then, quality assessment and proportion meta-analyses were performed.

**Results:**

The online search retrieved 233 articles and 15 were included for quantitative analysis. The risk of bias was low. The number of patients undergone Ctn testing was 29,533. The pooled percentage of those with ICV was 1.7% (95% confidence interval [CI]:1.2–2.3). The pooled proportion of MTC incidence among patients with ICV was 9.6% (95% CI:5–14.1). Heterogeneity was explained by the covariates of Ctn assay sensitivity and the resection rate. The subgroup with Ctn 10–20 pg/mL showed a significantly lower MTC rate than the subgroup with Ctn 20–100 pg/mL.

**Conclusions:**

The percentage of ICV among patients with thyroid nodules who underwent Ctn testing is negligible. The rate of MTC in patients with ICV cannot be overlooked. Among the ICV intervals, the risk of MTC increases significantly when Ctn is above 20 pg/mL.

**Supplementary Information:**

The online version contains supplementary material available at 10.1007/s11154-023-09811-7.

## Introduction

Medullary thyroid carcinoma (MTC) is a rare thyroid malignancy caused by C-cells that produce calcitonin (Ctn) [1]. Ctn measurement is recognized as the most accurate test to diagnose MTC, and MTC is associated with poor prognosis. Therefore, all patients with thyroid nodule(s) should be screened for this cancer by measuring the Ctn levels. Nevertheless, routine testing for Ctn levels is not universally accepted, and international recommendations in this context are ambiguous. Indeed, a consensus from the European Thyroid Association (ETA) in 2006 was in favor of routine Ctn testing during the initial nodule work-up [2]. The 2015 American Thyroid Association (ATA) guidelines were neither in favor nor against it [3]. And the 2010 ETA, American Association of Clinical Endocrinologists (AACE), and Associazione Medici Endocrinologi (AME) all recommended Ctn measurement in specific patient settings [4]. The lack of international consensus on this topic is mainly due to the fact that MTC occurs rarely, resulting that the cost-effectiveness of its universal screening is controversial. As reported by two relevant systematic reviews [5, 6], when encountering patients with newly discovered thyroid nodule(s), an MTC prevalence between 0.11% and 0.85%, with a median of 0.32%, can be expected. However, the matter is complicated, and whether this is a case of low cost-effectiveness merits careful clinical analyses. First, even if ultrasound (US) and US systems used in the risk stratification of thyroid nodules (i.e., Thyroid Imaging And Reporting Data System, TIRADS) represent the main method of diagnosing the nodules, it must be noted that their accuracy in detecting MTC is suboptimal [7]. Second, cytological examination of fine-needle aspiration (FNA) specimens is highly accurate for detecting papillary thyroid cancer, but it detects just more than half of MTCs [3, 8, 9]. Third, routine Ctn measurements can significantly improve the survival rate and prognosis of patients with MTC [10]. Fourth, a single Ctn test with a normal result may be a potential strategy to reduce costs [11]. Fifth, the cost of a Ctn test varies according to regional reimbursement laws [12]. From a purely clinical point of view, the lack of a fixed Ctn threshold to detect and exclude MTC and the wide range of indeterminate Ctn values (ICVs) (i.e., between the upper reference limit [generally 10 or 20 pg/mL] and 100 pg/mL) have hindered the universal acceptance and routine Ctn measurement tests in patients with thyroid nodule(s). In fact, ICVs may be due to both pathological and pathophysiological conditions (e.g., low-stage MTC, C-cell hyperplasia, renal disease, infection, and systemic inflammation), medications (e.g., proton-pump inhibitors), or lifestyle (e.g., smoking) [3]. Furthermore, it must be considered that Ctn varies according to individual features (i.e., sex and thyroid size), [3] and different normality references should be used. ICVs represent a challenge in clinical practice; there is no solid evidence-based data regarding its frequency and implications, and no clear recommendations, which can help clinicians to manage these cases. Thus, assessing the scope of this matter should encourage expert boards of international societies to create a standard for routine Ctn measurements.

This systematic review was designed to reappraise ICV comprehensively. Specifically, the study aimed to gather data on 1) the frequency of ICV among patients with thyroid nodule(s) who underwent the Ctn test and 2) the MTC rate among patients with ICV who underwent a full diagnostic work-up. A clinical discussion was conducted based on these findings.

## Methods

### Conduction of review

This systematic review was performed according to the Meta-analysis Of Observational Studies in Epidemiology guidelines [13]. (Supplemental Material—Table 1).

**Table 1:** General characteristics of the 15 included studies

**Ref**	**First author**	**Year**	**Journal**	**Country**	**Study period**	**Patients**	**Age (min–max)**	**Age (mean)**	**Ctn Assay**
[15]	Pacini	1994	J Clin Endocrinol Metab	Italy	1991	1385	13–78	N.R	IRMA
[16]	Rieu	1995	Clin Endocrinol (Oxf)	France	1989–1993	469	15–87	45	RIA
[17]	Shong	1996	J Kor Soc Endocrinol	Korea	1994–1995	1048	N.R	N.R	RIA
[18]	Vierhapper	1997	J Clin Endocrinol Metab	Austria	1994–1995	1062	N.R	N.R	RIA
[19]	Hahm	2001	Thyroid	Korea	1998–1999	1448	14–86	46	IRMA
[20]	Karanikas	2004	J Clin Endocrinol Metab	Austria	N.R	195	N.R	N.R	ICMA
[21]	Papi	2006	J Endocrinol Invest	Italy	2003–2004	1425	18–91	49.6	ICMA
[22]	Costante	2007	J Clin Endocrinol Metab	Italy	2001–2004	5817	11–72	49.7	ICMA
[23]	Herrmann	2010	Eur J Endocrinol	Germany	2005–2009	1007	41–69	55	ICMA
[24]	Schneider	2012	Nuklearmedizin	Germany	2004–2010	11,270	N.R	N.R	ICMA
[25]	Giovanella	2013	Clin Chem Lab Med	Switzerland	2008–2010	1236	36–70	53	ICMA
[26]	Turk	2017	Ann Surg Treat Res	Turkey	N.R	650	36–63	49.5	ICMA
[27]	Silvestre	2019	Eur Thyroid J	Portugal	2011–2015	1504	N.R	N.R	ICMA
[28]	Storani	2019	Medicina (B Aires)	Argentina	2009–2017	1017	18–88	56	ICMA
[29]	Rosario	2022	Horm Metab Res	Argentina	2021	N.R	N.R	N.R	ICMA

*N.R* not reported, *ICMA* immunochemiluminometric assay, *IRMA* immunoradiometric assay, *RIA* radioimmunoassay

### Search strategy

A five-step search strategy was planned: 1) sentinel studies were searched in PubMed; 2) keywords and Medical Subject Headings terms were identified; 3) electronic searches were performed on PubMed and Cochrane using the following terms: thyroid, calcitonin, and routine; 4) potentially eligible studies according to the authors were added to the screening; and 5) references of the included studies were screened for additional papers. The last electronic search was performed on January 27, 2023. No restrictions on publication year or language were imposed. Two investigators (TP and PT) independently, and in duplicate, searched for papers, screened titles and abstracts, reviewed full texts, and selected articles for inclusion. All discrepancies were resolved.

### Study selection

The inclusion criteria for the selected studies were: studies reporting a) data of cases with Ctn between 10–100 pg/mL (ICV) among thyroid nodule patients routinely screened for MTC by basal serum Ctn test and b) the number of MTC found among ICV patients. The exclusion criteria were as follows: a) using Ctn measurements only in nodules with suspicion of malignancy, b) measuring Ctn in individuals without thyroid nodules, c) studies with unclear data, and d) studies with overlapping data.

### Data extraction

The main papers of the included studies and supplementary data were searched. The following information was independently extracted from all included studies: (1) general study information (authors’ name and year of publication); (2) years included in the study; (3) kind of Ctn assay; (4) reference values of basal serum Ctn; (5) total number of patients evaluated; (6) patients with ICV; (7) patients with surgically treated ICV; (8) MTCs (pathology report) in patients with ICV; and (9) ICV attributed to medications, smoke, or chronic kidney disease. The relevant authors were contacted to request further data, when appropriate. The data were cross-checked, and discrepancies were discussed.

### Study quality assessment

The risk of bias in the included studies was assessed independently. The National Heart, Lung, and Blood Institute Quality Assessment Tool was used in this study. The following items were evaluated: study questions, eligibility criteria, sample size, description and delivery of the intervention, definition of outcome measures, duration of follow-up, blinding, loss to follow-up, and statistical methods. Each domain was assigned a low, high, or not reported score [14].

### Statistical analysis

A proportion meta-analysis could be performed to calculate 1) pooled proportion of patients with ICV among those consecutively screened (“ICVs/Patients”) and 2) pooled proportion of patients with pathologically proven MTC among those having ICV (“MTCs/ICVs”). In addition, further meta-analyses should be performed to estimate both ICV/patient and MTC/ICV rates in subgroups according to different ICV ranges (i.e., tertiles, quartiles, decades, etc.). All meta-analyses were performed when data from at least four studies were available. Heterogeneity was assessed by using I [2], and a value ≥ 50% detected presence of heterogeneity. A random-effects model was used. Pooled data were presented with 95% confidence intervals (95%CI). When heterogeneity was found, subgroup and meta-regression analyses were performed to explore the causes using several covariates (i.e., sample size, continent/country, duration and years of screening, case enrolment, age of population, Females/Males ratio, Ctn assay/upper reference limit, thyroid autoimmunity, selection criteria for surgery, resection rate, and rate of MTC). In the subgroup analysis, a significant difference was observed when the 95%CI of the two groups did not overlap. Statistical significance was set at p < 0.05. Statistical analyses were performed using Open Meta [Analyst] software (open-source software developed by the Center for Evidence Synthesis in Health, Brown University).

## Results

### Studies retrieved

**Fig. 1:**
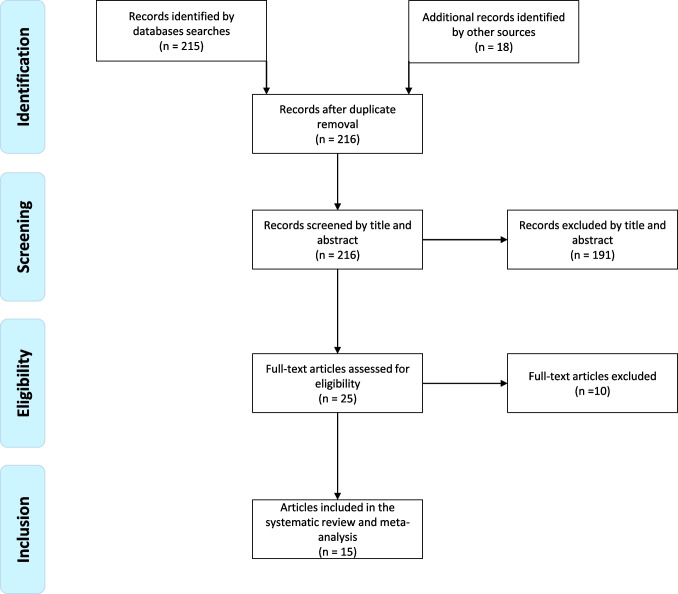
Flow of records found. Reason of exclusion of papers is reported in the text

A total of 233 records were identified using the search strategy. After removing duplicates and screening titles and abstracts, 25 papers were selected for full-text retrieval. Among these, 10 articles were excluded because of unclear data about ICV patients (n = 6) or since ICV cases were not counted (n = 4). Finally, 15 studies were included in this systematic review (Fig. 1).

### Study quality assessment

The risk of bias in the included studies is shown in supplemental material (Supplemental Material—Table 2). Overall, 10 of the 14 items were judged to have low risk of bias in all studies. Case selection (item 4) was assessed at an unclear risk of bias in eight studies because of the wide timing of case enrolment. Given the study design, the two items regarding the time-frame between exposure and outcome and the different levels or amounts of exposure were considered not applicable. No studies reported information regarding power or sample size justification.

**Table 2:** Data available in the 15 studies that allowed to perform meta-analysis

**Ref**	**First Author**	**Total number of cases screened for Ctn**	**Availability of data about MTC in ICV intervals**				
			**10–100**	**10–20**	**10–70**	**20–70**	**70–100**
[15]	Pacini	1385	✔	N.A	✔	✔	✔
[16]	Rieu	469	✔	N.A	N.A	N.A	✔
[17]	Shong	1048	✔	N.A	N.A	N.A	N.A
[18]	Vierhapper	1062	✔	✔	N.A	N.A	N.A
[19]	Hahm	1448	✔	✔	✔	✔	✔
[20]	Karanikas	195	✔	N.A	✔	N.A	N.A
[21]	Papi	1425	✔	✔	✔	✔	✔
[22]	Costante	5817	✔	✔	N.A	N.A	N.A
[23]	Herrmann	1007	✔	✔	✔	✔	N.A
[24]	Schneider	11,270	✔	✔	✔	✔	✔
[25]	Giovanella	1236	✔	✔	✔	✔	N.A
[26]	Turk	650	✔	N.A	N.A	N.A	N.A
[27]	Silvestre	1504	✔	N.A	N.A	N.A	N.A
[28]	Storani	1017	✔	✔	✔	✔	N.A
[29]	Rosario	N.A	✔	N.A	N.A	N.A	N.A

*ICV* indeterminate calcitonin value, *N.A* not available

### Qualitative analysis (systematic review)

The 15 papers included in this systematic review were published between 1994 and 2022. All were observational studies with retrospective data analysis at the end of the case enrollment period. Ten studies were published by European institutions (n.3 Italy, n.2 Austria, n.2 Germany, n.1 France, n.1 Portugal, and n.1 Switzerland), three were from Asia (n.2 Korea and n.1 Turkey), and the remaining two from Latin America (Argentina).

The total number of patients with thyroid nodules who underwent Ctn testing was 29,533. The study sample sizes ranged from 195 to 11,270 cases enrolled between 1989 and 2021. Patient ages ranged from 11 to 91 years. The study period ranged from 1 to 8 years (Table 1). Overall, 84 MTCs (2 in MEN 2A) were diagnosed histologically, with MTC percentages ranging from 0.1% to 0.8%.

Immunochemiluminometric assays (ICMA) were used in 10 studies, radioimmunoassay (RIA) in 3, and immunoradiometric assay (IRMA) in 2. Generally, the normal Ctn cut-off value was 10 pg/mL.

The total number of patients with ICV was 582.The ICV percentage was between 0.1% and 6.7%. The criteria used in the studies to indicate thyroidectomy in patients with ICV were quite heterogeneous (i.e., based on pentagastrin-stimulated Ctn > 100 pg/mL, FNA, and/or goiter-related symptoms). The resection rate among patients was between 5 and 100%. The number of histologically proven MTCs among patients with ICV was 42. Among the latter, no RET-mutated cases were found.

### Quantitative analysis (meta‐analysis)

**Fig. 2:**
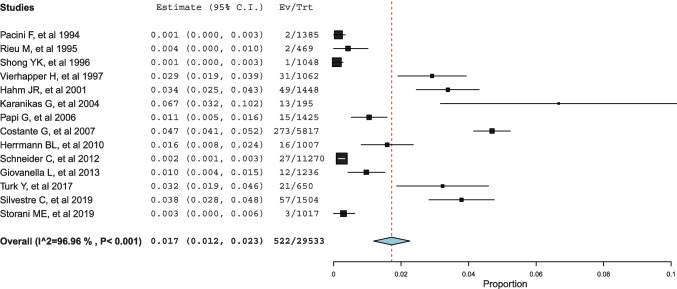
Forest plot meta-analysis of ICV among total number of patients screened for Ctn. Any square identifies the weight of the study. The diamond represents the pooled result and its wideness indicates 95%CI

**Fig. 3:**
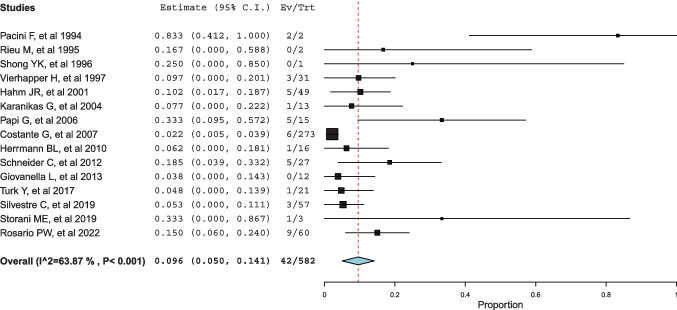
Forest plot meta-analysis of MTC recorded among ICV patients. Any square identifies the weight of the study. The diamond represents the pooled result and its wideness indicates 95%CI

The pooled percentage of ICV among all patients with thyroid nodules who underwent the Ctn test (ICVs/patients) was 1.7% (Fig. 2). According to Table 2, this calculation included 14 studies. The pooled proportion of MTC among patients with ICVs was 9.6% (Fig. 3).

**Fig. 4:**
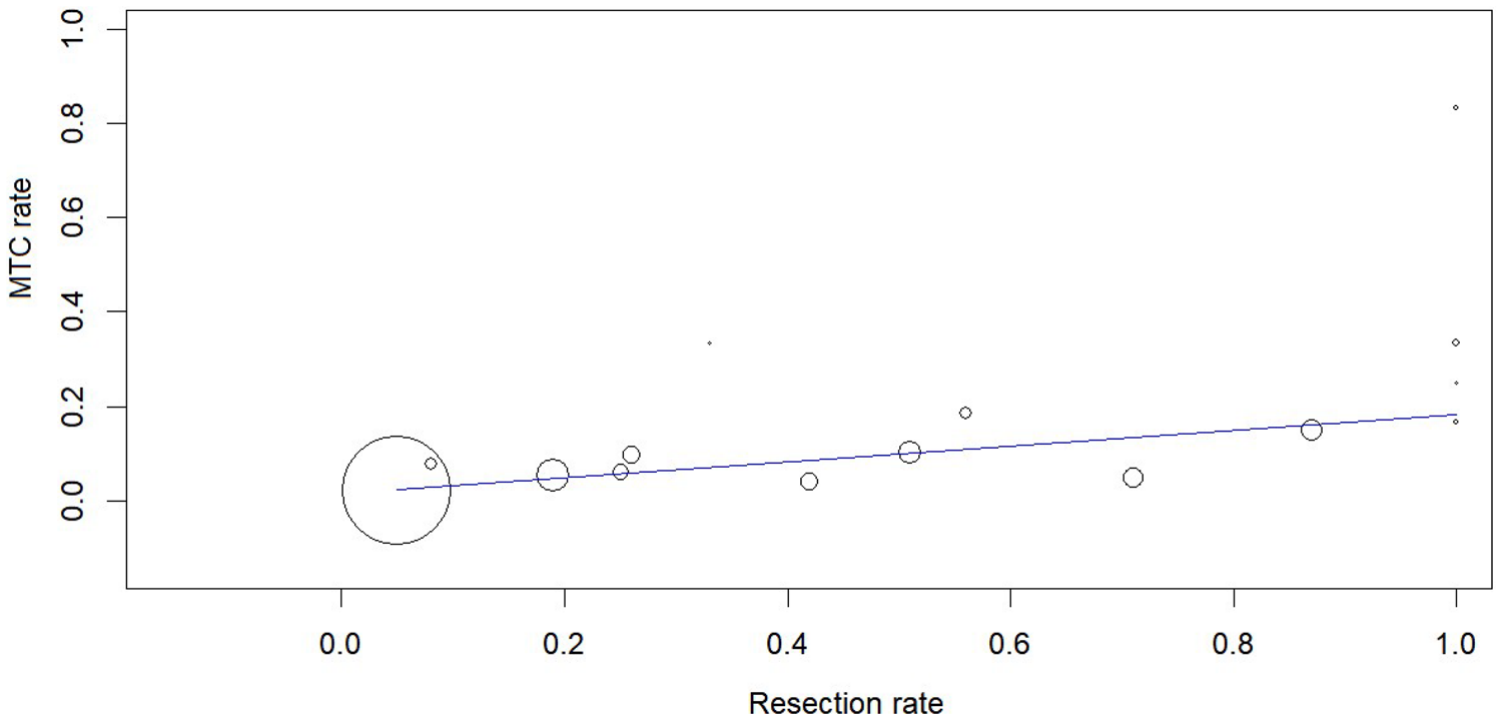
Meta-regression analysis using the covariate resection rate to explain the heterogeneous MTC rate among articles. Any circle identifies one study, and its size differs according to the study weight

The heterogeneity of both meta-analyses was explored according to the above covariates, when appropriate. Heterogeneity of the proportion of ICV among patients screened was solved with a meta-regression analysis using the covariate Ctn assay (p = 0.037) (Supplemental Fig. 1); the lower the Ctn assay’s analytical sensitivity, the higher the rate of ICV cases. Heterogeneity in the proportion of MTC among patients with ICV was explained by two meta-regression analyses using the covariates of Ctn assay sensitivity and resection rate. The higher the Ctn assay’s analytic sensitivity, the higher the MTC rate (p = 0.028) (Supplemental Fig. 2). The higher the resection rate, the higher the MTC rate (p < 0.001). Figure 4 illustrates the results of the most clinically relevant meta-regression analysis, and the supplemental material includes figures from other significant meta-regression analyses.

### Subgroup analysis according to different ICV intervals

This subgroup analysis aimed to evaluate the prevalence of MTC at different ICV intervals. According to the available data retrieved from the included studies (Table 2), 10 studies (19 MTCs among 317 ICVs) reported data that allowed for the calculation of the pooled MTC rate at three different ICV intervals.

**Fig. 5:**
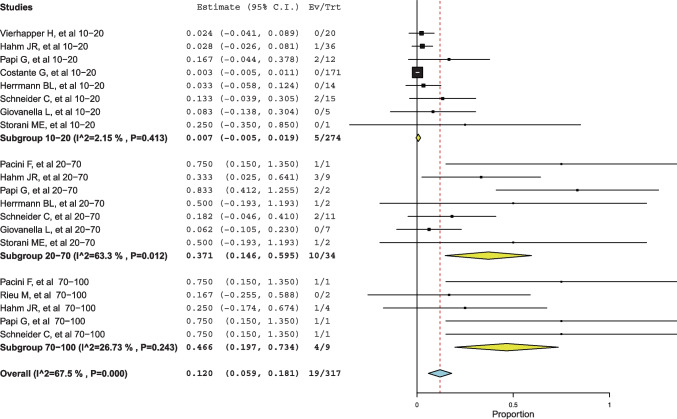
Forest plot meta-analysis of MTCs recorded within different ICV intervals. Any square identifies the weight of the study. The diamonds represent the pooled results and their wideness indicates 95% CI. The pooled MTC percentage of 12% derives from the included studies and differs from that recorded when including all studies

The three Ctn intervals were the following: 10–20, 20–70, and 70–100 pg/mL. According to these intervals, the pooled prevalence of patients was 1.2% (95%CI: 0.6–1.8; I^2^: 96.9%), 0.2% (95%CI: 0.1–0.3; I^2^: 49.8%), and 0.1% (95%CI: 0–0.1, I^2^: 43.5%), respectively. As illustrated in Fig. 5, the pooled percentages of MTC in the three intervals were 0.7%, 37.1%, and 46.6%, respectively. Because the 95% CI of the subgroup with Ctn 10–20 pg/mL did not overlap with that of the other subgroups, a significant difference was found.

## Discussion

MTC is a highly aggressive neuroendocrine thyroid tumor. Importantly, its prognosis is strongly correlated with timing of diagnosis, surgery being the only option to cure this cancer [30]. Ctn is recognized as the most reliable diagnostic marker of MTC, while US (and TIRADSs) and cytology can only detect just over 50% of the cases [1]. Unfortunately, other circulating markers as well as Ctn stimulation failed to significantly improve our diagnostic performance [1]. Despite the aforementioned information, there is no universal consensus regarding routine Ctn testing in patients with thyroid nodules. In absence of clear recommendations by international societies, the fear of facing patients with Ctn values elevated but not yet diagnostic for MTC (i.e., 10–100 pg/mL) may hold a role to discourage clinicians to measure Ctn in their patients. In fact, the management of patients with ICV must be clarified. Assessing both the risk of facing ICV and finding MTC among these patients can significantly help make clinicians aware and encourage them to use Ctn measurement assays.

This systematic review aimed to address these issues. Fifteen studies were retrieved, including 29,533 patients with thyroid nodules who underwent Ctn testing. The figures were relevant and findings obtained by meta-analyses can define the scope of the matter.

First, we considered all patients with thyroid nodule(s) screened using Ctn test; we found that the percentage of ICV was 1.7%. This measurement may depend on assay sensitivity. Second, the rate of MTC observed among patients with ICV was 9.6%. These results were significantly dependent on the resection rate (that is, the higher the operation rate, the higher the MTC number observed). Third, different Ctn subgroups of ICV were associated with different risk of MTC. The MTC rate found among patients with Ctn between 10 and 20 pg/mL was significantly lower than that observed among patients with Ctn > 20 pg/mL.

Overall, these data prompted a full discussion from a clinical standpoint. First, the fear of ICV is unjustified. In fact, more than 98% of patients have a Ctn level < 10 pg/mL, which is usually considered proof of absence of MTC, or > 100 pg/mL, which virtually diagnoses MTC. Moreover, the prevalence of patients having Ctn between 20 and 100 pg/mL was significantly lower than that of cases with Ctn between 10 and 20 pg/mL. Second, the risk of MTC cannot be overlooked and it increases with basal Ctn levels. Furthermore, the latter data may depend on institutional management and intrinsic features (that is, the experience of endocrinologists, surgeons, and cytopathologists) that influence the propensity to undergo surgery. This data must be considered in the context of the low accuracy of US and cytology, the pivot of thyroid nodule work-up, in detecting MTC [1].

Other issues were also discussed. First, the Ctn threshold, which is generally considered diagnostic for MTC, was 100 pg/mL. However, given the high percentage of MTC (~ 47%) in Ctn interval 70–100 pg/mL, this seems to be an arbitrary cut-off and may be revised. Therefore, we can also reduce Ctn stimulation tests since they are not supported by evidence-based data [1]. Second, the value of Ctn < 10 pg/mL represents virtual proof of the exclusion of MTC. Therefore, biochemical companies should adopt it as a standard reference. Third, high- and low-risk ICV exist (that is, 10–20 and 20–100 pg/mL, respectively). Since all of us address to surgery some (or a lot of) patients with indeterminate cytology that are associated with risk of malignancy of about 30% [31], the indication to diagnostic surgery in patients with high-risk ICV should be considered and fully discussed with patient. Those cases having Ctn between 20 and 100 pg/mL need full diagnostic work-up (i.e., FNA with cytology and Ctn measurement in aspiration fluids, Ctn stimulation test, measurement of other markers, neck US, other imaging procedures, etc.) and eventual surgery, while Ctn between 10 and 20 pg/mL could be managed with close and careful follow-up. Finally, the cost analysis should be mentioned. Even if the present data cannot allow to address this matter, in view of the findings we achieved, the cost of Ctn cannot represent a barrier. In fact, a single negative Ctn scan is sufficient to exclude the risk of MTC [11].

Although our systematic review included 15 studies with almost 30 k patients screened for MTC, there were some limitations. None of the included studies declared aims to evaluate patients with ICV; therefore, all data were extrapolated from papers with a different design with respect to our target population. Different Ctn assay kits were used, and different criteria were used to consider candidates for surgery. These two covariates best resolved the heterogeneity of the data collected. However, these discrepancies allowed us to fully explore the heterogeneity of the pooled data and thus achieve solid findings. Finally, publication bias cannot be excluded.

## Conclusions

Based on the results of the present systematic review and meta-analysis, we can conclude that: 1) the percentage of ICV among patients with thyroid nodule(s) who underwent the Ctn test is negligible (< 2%), 2) the rate of MTC observed among ICV patients cannot be overlooked (~ 10%), and 3) among ICV intervals, the risk of finding MTC increases significantly in the presence of Ctn > 20 pg/mL. Therefore, clinicians, and endocrinologists first, should be aware of these issues. It is necessary that international guidelines take a position on routine Ctn screening incorporating these concepts.

## Supplementary Information

Below is the link to the electronic supplementary material.

Supplementary file1 (PDF 34 KB)

Supplementary file2 (PDF 30 KB)

Supplementary file3 (DOCX 31 KB)

## Data Availability

The datasets generated during and/or analysed during the current study are available from the corresponding author on reasonable request.

## Abbreviations

MTC: Medullary thyroid carcinoma
Ctn: Calcitonin
US: Ultrasound
TIRADS: Thyroid Imaging and Reporting Data System
FNA: Fine-needle aspiration
ICV: Indeterminate calcitonin value
