# Supplementary figures and images for: Prevalence and significance of indeterminate calcitonin values in patients with thyroid nodules: A systematic review and meta-analysis
Source: Rev Endocr Metab Disord. 2023 May 31;24(4):685–94. doi: 10.1007/s11154-023-09811-7 (PMC10404572; doi:10.1007/s11154-023-09811-7)

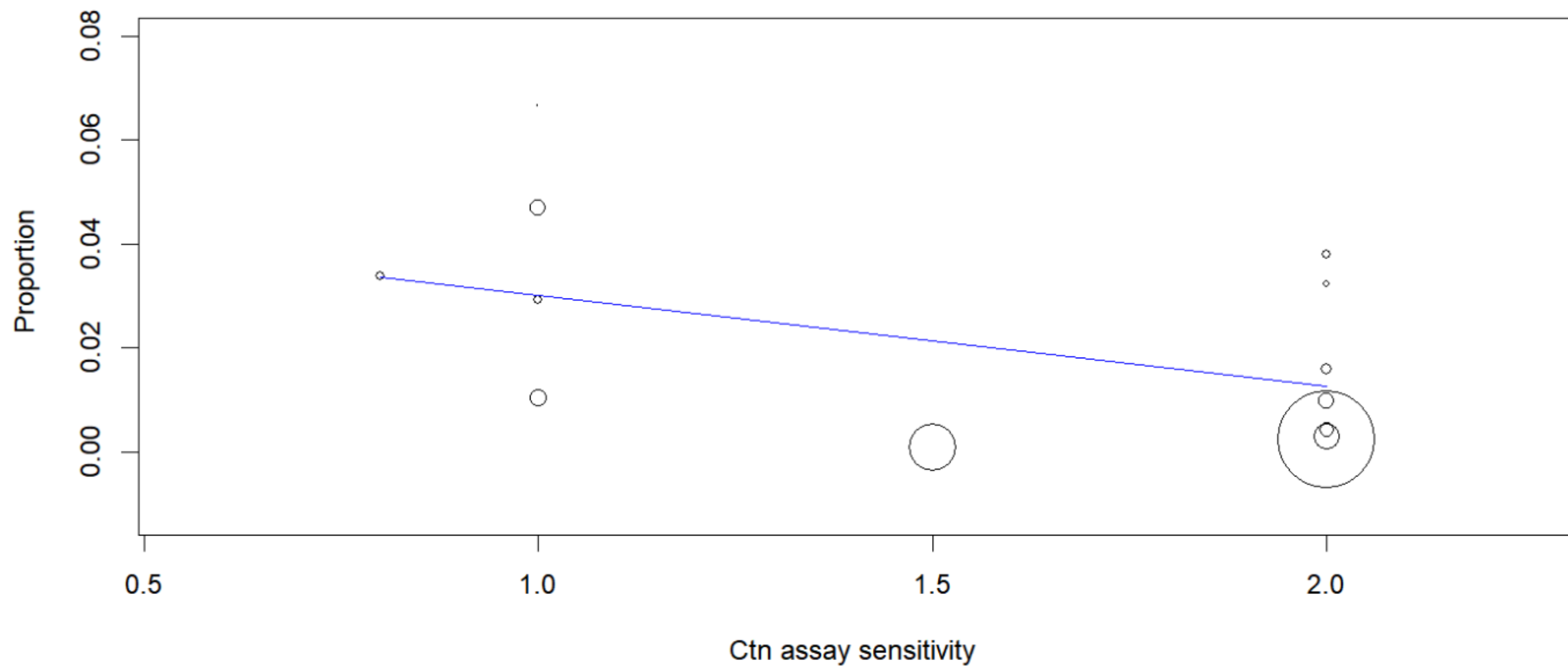

Supplement: Supplementary file 1 — Supplementary file1 (PDF 34 KB) [file 11154_2023_9811_MOESM1_ESM.pdf]

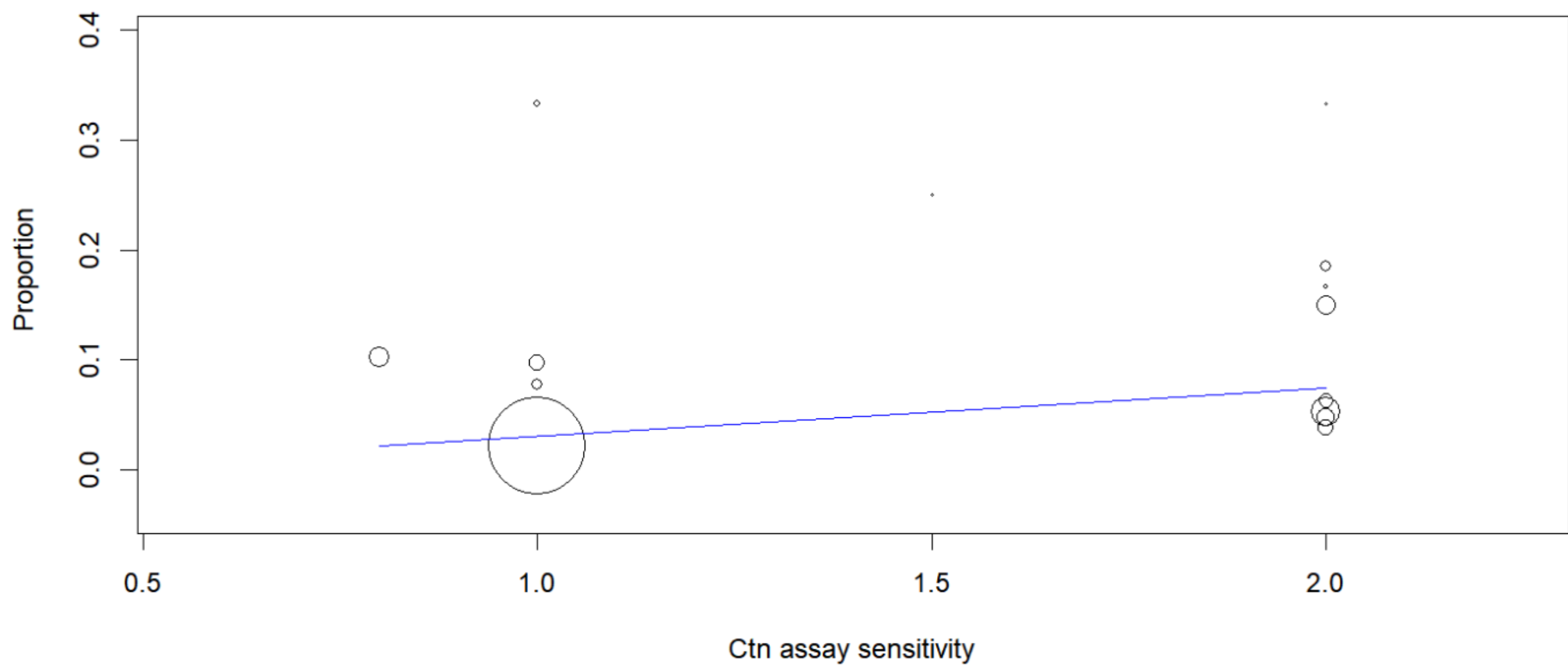

Supplement: Supplementary file 2 — Supplementary file2 (PDF 30 KB) [file 11154_2023_9811_MOESM2_ESM.pdf]
